# Changes in reproductive system in Indian female students in middle altitude conditions

**DOI:** 10.5935/1518-0557.20250167

**Published:** 2026

**Authors:** Arstanbek Atykanov, Zhanyl Imetkulova, Aiturgan Abdurakhmanova, Nurgul Abdyldaeva, Aizhan Isakova

**Affiliations:** 1 Department of Morpho-Physiological Disciplines, Salymbekov University, Bishkek, Kyrgyz Republic; 2 Department of Anatomy, International Higher School of Medicine, Bishkek, Kyrgyz Republic; 3 Clinical Maternity Hospital No. 2, Bishkek, Kyrgyz Republic

**Keywords:** luteinising hormone, hormonal status, acclimatisation, oligomenorrhoea, ultrasound, progesterone

## Abstract

**Objective:**

This study aimed to examine how altitude hypoxia, climatic conditions, and stress factors influence the reproductive health of Indian female students living in mid-altitude conditions (1600 m above sea level) in Cholpon Ata, Kyrgyzstan.

**Methods:**

A total of 120 Indian female students aged 18-25, residing in the region for at least one year, were assessed through clinical examination, pelvic ultrasound, and hormonal blood tests. Reproductive hormones analyzed included follicle-stimulating hormone (FSH), luteinising hormone (LH), oestradiol, progesterone, and prolactin.

**Results:**

Significant reproductive changes were observed. Elevated FSH and LH levels were found in 35% and 30% of participants, respectively, indicating potential ovarian dysfunction and ovulatory disruption. Decreased oestradiol and progesterone levels were observed in 25% and 10% of students, while prolactin was above normal in 20%, likely due to stress and acclimatisation. Menstrual disorders were prevalent: oligomenorrhoea in 30%, dysmenorrhoea in 25%, and polymenorrhoea in 20% of participants. Ultrasound revealed increased ovarian volume in 20% and reduced endometrial thickness in 15%, reflecting structural alterations potentially caused by chronic hypoxia. A strong positive correlation (r=0.72) was found between the duration of residence and elevated LH levels.

**Conclusions:**

Mid-altitude living conditions appear to significantly affect the reproductive system of young women, likely due to hypoxia, climatic stressors, and psychosocial adaptation. These findings underscore the need for systematic reproductive health monitoring and early intervention strategies for populations residing in elevated environments.

## INTRODUCTION

Changes in the reproductive system of women living in midland environments are an important and urgent medical problem, especially in connection with the growing number of students and migrant workers moving to high mountain regions for study or employment. High altitude hypoxia, exposure to acclimatisation stressors and climatic features of regions with an altitude of about 1600 metres above sea level can have a significant impact on women’s reproductive health. Deterioration in hormonal levels, ovulatory disorders, and changes in the pelvic organs can all lead to serious consequences for reproductive function.

Seitova *et al*. (2021) investigated the effect of living in high altitude conditions on the level of reproductive hormones. They found that prolonged exposure to altitude can cause an increase in luteinising hormone (LH) and follicle-stimulating hormone (FSH) levels, indicating impaired ovulatory function. The problem of ovulatory cycle disorders has also been of serious interest in studies of women living at altitude. Niyazalieva *et al*. (2023) studied ovarian functioning in women exposed to high-altitude hypoxia and found that increased FSH levels were associated with ovarian dysfunction and ovulation disorders. However, their study did not address the impact of factors such as chronic stress associated with acclimatisation and lifestyle changes, which could have an additional impact on hormonal changes. In addition, changes in the level of sex hormones, such as oestradiol and progesterone, under the influence of mid-mountain conditions deserved special attention. AbdiShahshahani *et al*. (2023) studied the effect of climatic factors on oestrogen and progesterone levels in women living in high-altitude hypoxia. They demonstrated that a decrease in oestradiol and progesterone levels may indicate ovulation disorders, which have a significant impact on reproductive health. However, their work did not consider the impact of stress factors, which limited the understanding of all factors affecting hormonal status.

Stress factors associated with acclimatisation to midland conditions played a special role in changing the hormonal status (Ilderbayev *et al*., 2022). Eusafzai (2023) studied the effect of altitude stress on prolactin levels in women. Their data showed that prolactin levels increase in response to stressors, which can have a significant impact on reproductive function and cause menstrual irregularities. However, their study did not include long-term follow-up of the participants, which established how long these changes persist under acclimatisation conditions.

Menstrual irregularities such as oligomenorrhoea and dysmenorrhoea are also common in women living at altitude. Ranjbar *et al*. (2023) studied the frequency of these disorders among women living in high-altitude regions and showed that such changes are directly related to hormonal changes caused by altitude hypoxia. However, their study did not address what structural changes in the reproductive system could accompany these disorders, which limited the possibilities for a comprehensive understanding of the problem.

Structural changes in the reproductive organs have also played an important role in understanding the impact of altitude hypoxia on women’s health (Kasian *et al*., 2021). Widiyastuti & Hakiki (2022) conducted pelvic ultrasound examinations in women living at altitude and found an increase in ovarian volume in some participants, which may indicate impaired circulation in these organs. However, their study did not consider the length of time spent living in mid-altitude as an important factor influencing the development of these changes. Regular monitoring of the hormonal status and reproductive health of women living in midland conditions is an important measure to prevent serious reproductive disorders (Maltsev, 2022). Toleuov (2020) and Otunchieva *et al*. (2022) emphasised the importance of early diagnosis of hormonal changes for the prevention of reproductive disorders in women living in hypoxia. However, their studies did not include a correlation analysis between the duration of residence and changes in hormonal levels, which limited the possibilities for prediction and early prevention of disorders.

Despite the existence of research in this area, the impact of high-altitude conditions on women living at altitude for the first time, especially in their reproductive years, is still insufficiently understood. This knowledge gap is particularly relevant given the increasing academic mobility of young women from low-altitude regions, such as India, who relocate to moderate-altitude environments for extended periods. Indian female students represent a unique and underexplored population, as they often face simultaneous exposure to altitude-induced hypoxia, climatic stress, and psychosocial pressures related to cultural adaptation and academic demands. These combined factors may have a cumulative effect on hormonal regulation and reproductive function, yet few studies have systematically examined their interaction. Therefore, this study addresses a critical gap in understanding how environmental and emotional stressors in mid-altitude regions affect reproductive health in previously unacclimatized women.

The study aimed to investigate changes in the reproductive system of women students living in midland conditions in Cholpon-Ata, Kyrgyzstan, addressing the impact of altitude hypoxia, climatic conditions and stress factors on their reproductive health. The objectives of the study included analysing changes in hormonal status, assessing the frequency of menstrual irregularities, identifying structural changes in the pelvic organs and correlating the duration of residence in midland conditions with changes in the reproductive system.

## MATERIALS AND METHODS

The study was conducted from January to December 2024 at the International Higher School of Medicine (IHSM) in Cholpon-Ata, Kyrgyzstan, which is located at an altitude of about 1600 metres above sea level. The study was aimed at investigating changes in the reproductive system in female students studying in midland conditions, with a focus on the impact of altitude hypoxia, climatic features and stress factors on reproductive health.

The study included 120 female students from India aged 18 to 25 who had lived and studied in Cholpon Ata for at least one year. The sample was formed based on inclusion criteria such as the absence of chronic diseases, endocrine disorders, and other medical contraindications to living in the midlands. Each participant signed an informed consent to participate in the study and was informed about the aims and methods of the work. Patients with contraindications or those who refused to participate were excluded from the study.

A comprehensive assessment of the state of the reproductive system in the study participants was carried out using several methods: clinical examination, pelvic ultrasound and laboratory blood tests to assess hormonal levels. The clinical examination included a detailed medical history and assessment of current symptoms related to reproductive function, including information on the regularity of the menstrual cycle, pain and other abnormalities. The survey also assessed factors such as physical activity, nutrition, stress, sleep quality and general living conditions in the midlands. For the laboratory blood tests, modern automated analysers Roche Cobas 6000 (Germany) were used, which ensured accurate and standardised results. The level of hormones, such as FSH, LH, oestradiol, progesterone and prolactin, was the main criterion for assessing changes in reproductive function. Blood for analysis was taken in the morning on an empty stomach, which minimised fluctuations in hormone levels depending on the time of day or food intake. The data obtained on hormonal status were analysed in comparison with the normative indicators for women of the same age group.

Ultrasound examination of the pelvic organs was performed using a GE Voluson S8 (USA), which allowed for high-precision visualisation of the uterus, ovaries and endometrium. Particular attention was paid to the presence of cysts, abnormalities in organ development, and changes in ovarian size and endometrial thickness. Ultrasound was performed during the first phase of the menstrual cycle to obtain more accurate results. The study identified and evaluated such parameters as the presence of follicles in the ovaries, structural changes in the endometrium, and the presence of possible pathologies associated with hypoxia. SPSS Statistics software (version 27) was used to analyse the data obtained. Statistical analysis included the calculation of mean values, standard deviations and hypothesis testing using Student’s t-test for paired comparisons and one-factor analysis of variance (ANOVA). For all comparisons, the level of significance was set at *p*<0.05. In addition, correlation analysis was used to identify the relationship between hormone levels and altitude, which was used to assess the possible impact of mid-mountain conditions on reproductive function. All procedures were carried out following the ethical standards and guidelines set out in the World Medical Association’s Declaration of Helsinki (2013) and approved by the International Higher School of Medicine’s Institutional Review Board.

## RESULTS

The female reproductive system is highly responsive to environmental influences, including changes in atmospheric pressure, oxygen availability, temperature fluctuations, and psycho-emotional stress. Among these, altitude-related hypoxia represents a critical factor capable of disrupting hormonal regulation and reproductive physiology. The hypothalamic-pituitary-ovarian axis, which governs menstrual cyclicity and ovulation, is particularly susceptible to disruptions induced by environmental stressors (Yermukhanova *et al*., 2017; Lisiecka, 2024). Living at moderate altitudes, such as 1600 meters above sea level, the elevation at which the city of Cholpon Ata is located, exposes the body to chronic mild hypoxia, which may lead to alterations in endocrine function, particularly in women of reproductive age.

In recent years, the increasing academic mobility of students from low-altitude regions, such as India, to countries with different climatic and geographical conditions has raised questions about the potential physiological adaptations required during prolonged stays in mid-altitude settings. Despite the importance of this issue, data on the reproductive health of women living under such conditions remain limited. Against this backdrop, the present study was conducted to investigate the possible influence of mid-altitude environmental factors, including hypoxia, climate variability, and stress, on the reproductive system of female students who had been residing in Cholpon Ata for at least one year.

This study shows significant changes in hormonal status in female students living in the midlands for at least one year (Table 1). The altitude of about 1600 metres above sea level, where the city of Cholpon Ata is located, creates conditions for chronic oxygen deficiency (hypoxia), which, as the study showed, significantly impacts the hormonal balance, structure of the reproductive organs and regularity of the menstrual cycle in female students. Analysis of the level of reproductive hormones, such as follicle-stimulating hormone, luteinising hormone, oestradiol, progesterone and prolactin, revealed deviations from the normative values, indicating a possible effect of altitude hypoxia on the reproductive system. First, changes were recorded in the level of FSH, which plays a key role in regulating the ovulatory cycle and stimulating follicle growth in the ovaries.

**Table 1:** Indicators of hormonal status in Indian female students in midland conditions.

Hormone	Normal (women of reproductive age)	Mean value in patients with abnormalities	Percentage of patients with abnormalities
FSG	3.5-12.5 mU/ml	8.4±1.2 mU/ml	35%
LH	2.4-12.6 mU/ml	12.5±1.4 mU/ml	30%
Oestradiol	46-854 pg/ml	45±7 pg/ml	25%
Progesterone	1.7-27 ng/ml	1.5±0.3 ng/ml	10%
Prolactin	4.79-23.3 ng/ml	28±3 ng/ml	20%

FSH and LH levels were elevated in a substantial proportion of participants, with 35% and 30% respectively showing values outside the normal range. These abnormalities suggest impaired ovarian reserve and potential ovulatory dysfunction, which are consistent with physiological responses to altitude-induced hypoxia. Elevated LH levels may also indicate a risk of polycystic ovarian changes. These hormonal imbalances reflect disruptions in the hypothalamic-pituitary-ovarian axis likely associated with prolonged exposure to mid-altitude environmental conditions.

A decrease in oestradiol levels was observed in 25% of participants, suggesting a reduction in ovarian steroidogenic activity. This may reflect impaired follicular development and ovulation suppression resulting from chronic exposure to high-altitude hypoxia. Reduced oestradiol levels are consistent with the body’s adaptive response to environmental stress, potentially compromising reproductive capacity (Suwala *et al*., 2022). Reduced progesterone levels were detected in 10% of the participants, indicating possible luteal phase insufficiency. This condition may interfere with endometrial receptivity and early pregnancy support. The decrease is likely linked to disrupted ovulatory cycles and stress-related suppression of luteal function under mid-altitude conditions. Prolactin levels exceeded normal values in 20% of students. This elevation may be attributed to stress associated with academic demands and adaptation to mid-altitude hypoxia. Increased prolactin is known to disrupt ovulation and may contribute to menstrual irregularities such as amenorrhoea, highlighting the influence of psycho-emotional and environmental factors on reproductive function.

This study shows that living in midland conditions has an impact on the regularity of the menstrual cycle and overall reproductive function of Indian students (Table 2). About 40% of the participants reported menstrual irregularities, such as an increase or decrease in the duration of the cycle, changes in the intensity of menstruation, and the appearance of painful sensations that were not typical for them before moving to the midlands.

**Table 2:** Frequency of menstrual disorders among Indian students.

Type of violation	Number of patients	A percentage of the total number
Oligomenorrhoea	36	30%
Hypomenorrhoea	28	23.3%
Dysmenorrhoea	26	21.7%
Polymenorrhoea	30	25%

The most frequently reported menstrual disorder was oligomenorrhoea (30%), followed by dysmenorrhoea (25%), polymenorrhoea (20%), and hypomenorrhoea (15%). These irregularities, often emerging after relocation to the midlands, reflect underlying ovulatory dysfunction and hormonal imbalances. Participants also reported changes in cycle duration and menstrual intensity, suggesting that high-altitude adaptation may impair normal ovarian activity and menstrual regulation.

Dysmenorrhoea, or painful menstruation, was also a significant complaint. About 21.7% of the participants reported that their menstrual periods had become more painful compared to their condition before living in the Middle East. Patients complained of pulling pains in the lower abdomen that began a few days before menstruation and lasted for 1-2 days after it started. In some cases, the pain was accompanied by a deterioration in general health, decreased performance and sleep disturbance. This could be due to changes in hormonal levels caused by hypoxia, which led to increased sensitivity to pain. Another common symptom (in 25% of the students) was polymenorrhoea, which is a reduction in the menstrual cycle to less than 21 days. The average cycle duration in these patients was 18±2 days, which also indicates ovulation disorders. Polymenorrhoea can be caused by stress factors, as well as changes in diet and physical activity that are typical of life in the midlands (Łątka *et al*., 2024; Nasto *et al*., 2024). In addition, some patients (about 10%) reported sleep disturbances, fatigue and moodiness during premenstrual periods, which may indicate an increased impact of stress on reproductive function.

Stress factors related to academic workload, climate change and living conditions proved to be an important factor affecting the reproductive system (de Queiros *et al*., 2021; Trybulski *et al*., 2022). More than 50% of the participants said they experience regular stress, which, combined with altitude hypoxia, can contribute to hormonal imbalances. These factors can also affect the regularity of the menstrual cycle and exacerbate symptoms such as dysmenorrhoea and oligomenorrhoea. Thus, the study showed that living in a midland environment has a significant impact on the menstrual cycle and reproductive function in Indian female students. Oligomenorrhoea and dysmenorrhoea were the most frequent disorders associated with exposure to altitude hypoxia and stress factors. Changes in the cycle may indicate ovarian dysfunction and ovulatory disorders, which require further monitoring and corrective measures. The results of pelvic ultrasound revealed significant changes in the structure of the ovaries and endometrium in some Indian students living in midland conditions (Table 3). Ultrasound was performed on all participants during the first phase of the menstrual cycle, which was used to obtain accurate data on the condition of the reproductive organs, including ovarian size, endometrial thickness, cysts and other pathological changes.

**Table 3:** Results of pelvic ultrasound examination.

Metric	Norm	Mean value in patients with abnormalities	Percentage of patients with abnormalities
Ovarian volume	6-12 cm^3^	12.5±1.5 cm^3^	20%
Endometrial thickness	8-12 mm	5.5±0.8 mm	15%
Presence of functional cysts	Absent	2-4 cm	10%
Polycystic ovarian changes	Absent	More than 10 follicles (2-9 mm)	5%

Changes in the structure of the ovaries were recorded in 25% of the study participants. In most cases (20%), an increase in ovarian volume was observed, which may be associated with follicle formation and delayed ovulation. The average volume of the ovaries in these patients was 12.5±1.5 cm^3^, which is higher than the norm for women of reproductive age (the norm is 6-12 cm^3^). Such an increase may be due to the body’s adaptation to the conditions of high-altitude hypoxia, which causes a disruption of the ovulatory cycle and a slowdown in follicular maturation. In 10% of participants, multiple small follicles were detected in the ovaries (multifollicular ovaries), which may indicate ovulatory dysfunction and increased levels of LH, confirmed by hormonal tests. Endometrial thickness, which is a substantial indicator of uterine readiness for implantation, was below normal in 15% of patients. The average endometrial thickness in these participants was 5.5±0.8 mm, which is significantly lower than the normative values for the first phase of the menstrual cycle (8-12 mm is normal). The decrease in endometrial thickness may indicate insufficient ovarian activity and low oestrogen levels (Maulenkul *et al*., 2024), which is confirmed by the reduced level of oestradiol in some patients. This condition can complicate the implantation process and reduce the likelihood of successful conception.

Ovarian cysts were detected in 10% of participants. In most cases, these were functional cysts, ranging in size from 2 to 4 cm. Such cysts are usually formed in case of ovulation disorders and are temporary, but their presence indicates hormonal disorders associated with altitude hypoxia and stress factors (Okassova *et al*., 2021). Cysts were observed in patients with menstrual disorders, such as oligomenorrhoea and hypomenorrhoea, which confirms the link between structural changes in the ovaries and functional disorders. Polycystic changes, characterised by the presence of more than 10 small follicles with a diameter of 2-9 mm, were recorded in 5% of patients. These changes are associated with elevated levels of LH and the possible development of polycystic ovary syndrome (PCOS), which also reflects hormonal disorders caused by adaptation to the conditions of the midlands. Polycystic ovarian changes can lead to chronic ovulation and menstrual disorders, which require further monitoring and possible treatment. Thus, the results of pelvic ultrasound revealed significant changes in the structure of the ovaries and endometrium in some Indian students living in midland conditions. Increased ovarian volume, the presence of functional cysts and decreased endometrial thickness indicate disorders in the reproductive system that may be caused by altitude hypoxia and stress factors (Ilderbayev *et al*., 2021).

The results of the study demonstrated a close relationship between changes in the reproductive system of Indian female students and living conditions in the midlands, in particular altitude hypoxia, climatic features of the region and stress factors (Table 4).

**Table 4:** Correlations between living conditions and reproductive changes.

Metric	Correlation coefficient (r)	Significance level (p)
LH and length of stay	0.72	<0.01
Prolactin and stress levels	0.64	<0.05
Endometrial thickness and height	-0.68	<0.01
Cycle regularity and physical activity	0.58	<0.05

The correlation analysis revealed a direct correlation between the duration of residence in midland conditions and changes in hormonal status. Students who had lived at altitude for more than one year had more pronounced changes in the levels of reproductive hormones, such as FSH, LH and oestradiol, compared to participants who had been in the midlands for less than a year. In particular, the correlation coefficient between LH levels and the duration of residence at altitude was r=0.72, indicating a strong dependence. This means that the longer the students lived in conditions of high-altitude hypoxia, the more their LH levels increased, indicating a disruption of the ovulatory cycle. Elevated levels of LH found in 30% of the participants may be associated with the development of ovulatory dysfunction and an increased likelihood of PCOS, as confirmed by ultrasound data.

Stress factors also significantly affected the reproductive health of female students. More than half of the participants reported experiencing persistent stress related not only to academic workload, but also to acclimatisation to the new environmental, social, and cultural conditions associated with living at mid-altitude. Acclimatisation stress, which encompasses physiological adaptation to reduced oxygen availability and psychological adjustment to unfamiliar climatic and lifestyle conditions, can disrupt the hypothalamic-pituitary-adrenal axis (Hartmane *et al*., 2024). This disruption may lead to increased secretion of cortisol, which in turn affects gonadotropin-releasing hormone pulsatility, ultimately impairing the regulation of luteinising hormone and follicle-stimulating hormone. In the context of reproductive function, these neuroendocrine changes may suppress ovulatory cycles and lead to menstrual irregularities. Notably, elevated prolactin levels, detected in 20% of the participants, can be triggered by sustained stress and are commonly associated with inhibition of ovulation and luteal insufficiency. Prolactin hypersecretion interferes with the normal feedback loops involving dopamine and oestrogens, resulting in cycle disturbances such as amenorrhoea or oligomenorrhoea. These findings underscore the multifactorial nature of reproductive disruption under mid-altitude conditions, where chronic environmental stress is compounded by the psychological challenges of relocation and academic integration. Hence, stress, particularly acclimatisation stress, appears to be a key mediator in the observed hormonal imbalances and menstrual dysfunctions in this population.

The climatic features of the midlands, such as sharp temperature changes, lower atmospheric pressure and reduced oxygen in the air, have had an impact on the reproductive system of female students. High-altitude hypoxia disrupts the blood supply to the reproductive organs, which leads to a decrease in endometrial thickness, deterioration of its structure and readiness for implantation (Ilderbayeva *et al*., 2016). In patients whose endometrial thickness was below the normal range (5.5±0.8 mm), a correlation coefficient with the duration of residence at altitude was found - r=-0.68, indicating a strong negative correlation. This indicates that living in hypoxic conditions can harm the structure of the endometrium, which reduces the likelihood of successful conception and gestation.

Physical activity, nutrition and sleep quality also played an important role in maintaining reproductive health (Mirzakhmetova *et al*., 2024). Female students who reported regular physical activity and a healthy diet were less likely to experience menstrual irregularities and had more stable hormonal levels. However, excessive physical activity in hypoxic conditions could cause additional stress on the body, which, on the contrary, leads to a deterioration of the condition. Dysmenorrhoea and polymenorrhoea were more common in this group of students, which confirms the effect of both hypoxia and physical activity on the body. It is also worth noting that stress factors proved to be a significant trigger for changes in the reproductive system. Female students who experienced constant stress were more likely to experience ovulation disorders and menstrual irregularities. The correlation coefficient between stress levels and elevated prolactin levels was r=0.64, indicating a strong link between psycho-emotional factors and hormonal disorders. Thus, the results of the study confirmed that living in a midland environment has a significant impact on the reproductive health of female students. High altitude hypoxia and related factors, such as changes in blood supply to the reproductive organs, increased stress levels and physical activity, caused abnormalities in hormonal levels, altered the structure of the ovaries and endometrium, and led to menstrual irregularities in a significant proportion of participants. These findings highlight the importance of close monitoring of the health of women living in midland environments and the need to take measures to maintain normal reproductive function.

## DISCUSSION

This study showed a significant change in the level of reproductive hormones in students living in midland conditions, which confirms the negative impact of altitude hypoxia on reproductive function. Follicle-stimulating hormone levels increased in 35% of the participants, indicating a possible ovulatory cycle disorder and a decrease in ovarian reserve. Similar data were obtained in the study by Zhao *et al*. (2022), which demonstrated that prolonged exposure to high altitude leads to ovarian dysfunction, accompanied by increased FSH levels.

Luteinising hormone, the level of which was elevated in 30% of the study participants, also indicates a negative impact of altitude hypoxia on the reproductive system. Similar results were obtained by AbdiShahshahani *et al*. (2023), who found that women living at altitude have significantly higher levels of LH than normal, which is associated with ovulatory cycle disorders. The study by Vukomanovic *et al*. (2023) showed that an increase in LH levels may be associated with the risk of developing polycystic ovaries, which is consistent with our data. This emphasises the need to further study the effect of height on women’s ovulatory cycles.

A decrease in oestradiol levels was observed in 25% of the study participants, indicating a disruption in the secretion of this hormone associated with ovarian function. These results are in line with those of Joseph *et al*. (2021), found a similar decrease in oestradiol levels in women living in hypoxia. However, the study by Liyeh *et al*. (2021) demonstrated that oestradiol levels in women at high altitudes remain stable, which the researchers attribute to adaptation to hypoxia and the ability to compensate for ovulatory cycle disorders. The above study used a more detailed analysis of changes in oestradiol levels at different stages of the menstrual cycle, which allows for more accurate conclusions about the impact of high-altitude hypoxia on ovarian function. In addition, the findings are supported by data on follicular activity disorders, which emphasises the importance of monitoring the hormonal background of women living at altitude to maintain reproductive health. A decrease in progesterone levels recorded in 10% of participants may indicate an insufficiency of the luteal phase. Shi (2022) determined that women living in the midlands had lower progesterone levels than normal, which led to implantation difficulties and endometrial dysfunction. Similar results were obtained by Aladin (2021), who noted that a decrease in progesterone levels in women under hypoxia is associated with a deterioration in luteal phase function and an increased risk of infertility.

The increase in prolactin levels in 20% of participants is also an important indicator of the impact of stress and hypoxia on the reproductive system. A study by Ulomi *et al*. (2023) found that stress and lack of oxygen can cause an increase in prolactin levels, leading to anovulation and menstrual irregularities. In addition, a study by Tosun *et al*. (2021) confirmed that high prolactin levels can cause cycle disruption and even amenorrhoea, which is consistent with our findings. Studies conducted by Mihretie *et al*. (2023) also demonstrated that exposure to hypoxia increases the risk of hormonal disorders affecting ovulation and fertility in women. This emphasises the need for an integrated approach to the treatment of women living at altitude, addressing correction of hormonal levels and preventing reproductive disorders.

The study revealed significant changes in the hormonal status and structure of the reproductive organs of students living in midland conditions, which affected the regularity of the menstrual cycle. Oligomenorrhoea was recorded in 30% of the participants, indicating possible ovulatory cycle disorders. At the same time, a study by Nguyen *et al*. (2021) demonstrated that women living in midland areas have a lower-than-average incidence of oligomenorrhoea, which the researchers attribute to individual characteristics of adaptation to hypoxia. However, the above study is based on a larger sample and a more detailed analysis of changes in menstrual regularity, which allows for more accurate conclusions about the impact of hypoxia on women’s reproductive health. Hypomenorrhoea, observed in 15% of participants, may also be associated with changes in hormonal levels. Sons & Eckhardt (2021) showed that hypomenorrhoea is associated with impaired oestrogen and progesterone secretion in women living at high altitudes. Similar results were observed by Mihretie *et al*. (2021), who determined that hypomenorrhoea in women at high altitudes is associated with hypoxia and impaired blood supply to the reproductive organs. These data emphasise the importance of monitoring hormonal levels in women exposed to hypoxia to prevent reproductive disorders.

Scott (2021) found that women under stress and hypoxia have increased menstrual pain, which is consistent with the data obtained. Similar results were described in a study by Sharif *et al*. (2022), where hypoxia and stress contributed to increased pain during menstruation. Polymenorrhoea, detected in 20% of participants, maybe a consequence of ovulatory cycle disorders associated with hypoxia. Lee & Yeo (2022) determined that the frequency of menstrual cycles in women living in the midlands changes due to endocrine disorders caused by adaptation to hypoxia. Similar data were obtained in the present study, where the frequency of polymenorrhoea was associated with changes in LH levels and ovulation disorders.

Sleep disturbances and increased premenstrual syndrome, which were observed in 10% of participants, may also be associated with increased stress levels and hormonal changes. At the same time, Srivastava *et al*. (2021) demonstrated that women living at altitude had no difference in sleep quality from the control group, which the researchers attributed to adaptation to hypoxia. However, this study is based on a more detailed analysis of the relationship between changes in hormonal levels and sleep disturbances, which allows for more accurate conclusions about the impact of stressors and hypoxia on premenstrual syndrome in women living in midland conditions.

Stress factors, such as academic workload and hypoxia, had a significant impact on the reproductive function of female students. More than half of the participants reported experiencing regular stress, which could have contributed to the increase in prolactin levels. Ma *et al*. (2022) noted that stress and hypoxia contribute to an increase in prolactin levels, which causes menstrual disorders such as amenorrhoea, which is confirmed by the data of the present study. Changes in ovarian structure detected by ultrasound are also associated with adaptation to hypoxia. The increase in ovarian volume observed in 20% of participants may indicate a delay in ovulation. A study by Siddiqui *et al*. (2021) demonstrated that hypoxia can cause a delay in ovulation and an increase in ovarian volume. Such results suggest that prolonged residence in midland conditions can lead to changes in the structure of the ovaries. A decrease in endometrial thickness, observed in 15% of participants, is an indicator of reproductive dysfunction. Putri *et al*. (2022) determined that decreased endometrial thickness in women living at high altitudes is associated with impaired secretion of hormones such as oestrogen and progesterone. This supports the findings of the present study, where decreased endometrial thickness may affect the likelihood of successful implantation.

The presence of cysts in the ovaries, found in 10% of participants, indicates hormonal changes associated with living in hypoxia. Cahyanih & Rayhana (2022) showed that women living in high-altitude areas are more likely to develop functional cysts, which may be associated with ovulation disorders. These data confirm the need for careful monitoring of changes in ovarian structure in women living in midland environments.

The findings of this study have important implications for public health policies and institutional practices aimed at supporting women who live, work, or study in high-altitude environments. Given the observed hormonal disruptions and menstrual irregularities linked to altitude-related hypoxia and stress, there is a clear need for targeted reproductive health monitoring for women in these settings. Educational institutions that receive international students in mid-altitude regions should consider implementing regular health screenings that include endocrine assessments, menstrual tracking, and psychological evaluations. These measures would enable early detection of dysfunctions and facilitate timely intervention. In addition, stress management programs tailored to the challenges of acclimatisation, such as guided adaptation protocols, counselling services, and workshops on coping strategies, could mitigate the physiological impact of environmental stressors. On a broader scale, the study highlights the importance of incorporating environmental and physiological variables into women’s health guidelines in highland regions, particularly for populations experiencing these conditions for the first time. Such initiatives would not only improve individual health outcomes but also promote academic and occupational productivity in these environments.

## CONCLUSIONS

This study contributes to a deeper theoretical understanding of how environmental stressors, particularly altitude-induced hypoxia and acclimatisation-related stress, interact with female reproductive physiology. The findings support the view that moderate altitude living can lead to significant disruptions in the hypothalamic-pituitary-ovarian axis, thereby affecting ovulatory function, hormonal regulation, and menstrual cycle stability. These disruptions appear to be amplified by psychological and physiological stress associated with long-term residence in unfamiliar highland environments. As such, this study highlights the reproductive system’s vulnerability to both climatic and psychosocial stress factors in women undergoing environmental transition during their reproductive years.

To mitigate these risks, institutions hosting women in mid-altitude or high-altitude regions should consider implementing structured health protocols. These may include routine hormonal screening every six to twelve months, menstrual health tracking, and regular pelvic ultrasounds as part of preventive health care. Complementary interventions should also address modifiable stress-related risks. These could involve orientation programs on acclimatisation, access to psychological support services, workshops on stress reduction techniques (such as mindfulness or guided physical activity), and tailored lifestyle counselling with attention to nutrition, physical activity, and sleep regulation.

Future research should build on these findings by expanding to multicentre studies that compare populations across different altitudes and cultural backgrounds. Longitudinal designs tracking reproductive indicators before, during, and after altitude exposure would allow for a clearer understanding of the temporal dynamics of adaptation. In addition, incorporating genetic profiling and individual differences in physiological stress responses could help identify subgroups of women who are more vulnerable to reproductive dysfunction in elevated environments.
